# Reality stabilization: a unified framework for psychosis and post-psychotic depression

**DOI:** 10.3389/fpsyg.2026.1805102

**Published:** 2026-10-14

**Authors:** Georgi Panov, Presiyana Panova

**Affiliations:** 1 Psychiatric Clinic, University Hospital for Active Treatment "Prof. Dr. Stoyan Kirkovich", Trakia University, Stara Zagora, Bulgaria; 2 Department of Neurology, Psychiatry and Psychology, Government University "Prof d-r Asen Zlatarov", Burgas, Bulgaria; 3 Medical Faculty, Trakia University, Stara Zagora, Bulgaria

**Keywords:** cognition, default mode network, depression, dopamine, psychosis, quantum cognition, reality stabilization, schizophrenia

## Abstract

Both quantum mechanics and cognitive neuroscience challenge the classical view of a passively perceived reality. Instead, they suggest that reality arises through a process of selection and stabilization from a field of possibilities. This article proposes that this “reality stabilization” framework can unify our understanding of psychosis and post-psychotic depression—two conditions that are currently studied as distinct phenomena, and which existing models of predictive coding, dopaminergic dysregulation, and network neuroscience rarely integrate into a single account. We conceptualize psychosis as a failure of neurocognitive decoherence, in which competing perceptual and interpretative states cannot be reduced to a single, coherent reality. Post-psychotic depression, in contrast, is framed as a compensatory hyperstabilization, in which a rigid, negatively valenced reality is restored, possibly mediated by altered DMN activity. The framework suggests that effective intervention must move beyond dopaminergic blockade to include strategies that promote adaptive network coherence and cognitive integration. The model remains hypothetical, and direct empirical evidence for decoherence-like processes, network changes, and the proposed temporal progression is limited. Future work should focus on empirical testing of stabilization and hyperstabilization mechanisms, longitudinal assessment across illness stages, and their possible therapeutic relevance.

## Introduction

Classical intuition presumes that reality is a given entity, passively perceived by an observer. However, this view has been fundamentally challenged in two seemingly disparate fields: quantum physics and cognitive neuroscience. In quantum mechanics, the act of measurement does not create a new reality ex nihilo but constrains a pre-existing set of possibilities into a single, stable state—a process known as decoherence (Zurek, 2003; Schlosshauer, 2007). The outcome is not arbitrary but selected through interaction, yielding a “collapsed” and workable reality from a superposition of potentialities. By “state” we mean a potential interpretation, perception, or cognitive configuration; by “system” we mean the cognitive apparatus as a whole; and by “decoherence” we mean the process by which competing possibilities are reduced to a single, stable subjective reality. The act of “observation” (conscious attention) can no longer be separated from the state of the system, leading to an irreversible selection of reality (Atmanspacher, 2004; Bruza et al., 2015).

This structural analogy is supported by recent work in quantum cognition research. Classical probability theory is often insufficient to model cognitive phenomena such as order effects, context dependence, and ambiguity, and the quantum formalism offers a mathematically rigorous alternative (Bruza et al., 2015; Pothos and Busemeyer, 2013). Furthermore, Xiong and Yu (2025) argue that the superposition state of quantum systems is a condition for the emergence of consciousness, and that the function of consciousness is precisely to process these superposition states, leading to their collapse. This directly parallels our framework: conscious attention selects among competing interpretations of reality, reducing a field of possibilities to a single, stabilized state. We present this parallel as a structural analogy, not as a claim of literal physical equivalence. Whether quantum and cognitive processes might reflect a deeper ontological continuity is a separate empirical question that lies beyond the scope of this article and does not affect the framework presented here.

A conceptually parallel process underpins the construction of subjective reality in the human mind. The brain does not passively receive sensory data but actively generates predictions and models of the world, continuously selecting and stabilizing a coherent perceptual and narrative experience from a flux of internal and external signals (Friston, 2010). This stabilization is crucial for adaptive functioning. Within the framework of predictive coding, the key mechanism for this selection is the weighting of precision (or certainty) assigned to sensory inputs versus prior beliefs (Feldman and Friston, 2010). Precision weighting acts as the brain’s arbitrator, determining which signals dominate consciousness and which are suppressed, thereby guiding the “collapse” of subjective experience into a stable, unified reality. The neural implementation of this process involves large-scale networks. Menon’s (2011) triple network model proposes that the Salience Network (SN) acts as a dynamic switch between the Default Mode Network (DMN) and the Central Executive Network (CEN). Empirical data further demonstrate that the structural and functional connectivity across these core neurocognitive brain networks undergo dynamic reconfigurations during development (Seeley et al., 2007; Uddin et al., 2011). We propose that this SN-mediated switching is functionally analogous to adaptive precision weighting—prioritizing internal predictions (DMN) versus sensory evidence (CEN). This is an interpretive extension of Menon’s model, not a direct claim from the cited literature (Menon, 2011; Seeley et al., 2007; Uddin et al., 2011).

In essence, this ongoing process of selection, integration, and stabilization is cognition at its most fundamental level. We define “core cognitive functions” as attention (the focusing of precision on selected signals), working memory (the temporary maintenance of a stabilized state), and long-term memory (the consolidation of that state into a prior belief for future use). These are all mechanistic components of this reality-construction apparatus. We define “adaptive cognition” as the efficient, context-sensitive stabilization of a workable subjective reality from the noisy flux of potential experiences. This definition builds on existing accounts of cognitive flexibility and predictive processing (Friston, 2010; Diamond, 2013).

Critically, the space of possibilities from which this stable reality is selected is not neutral or universal. It is fundamentally shaped by individual history—by prior experience. Early, especially adverse or fragmented experiences, particularly during sensitive developmental periods, can durably alter the neurocognitive architecture that performs this selection (Hensch, 2005; Knudsen, 2004). Such experiences may restrict integrative capacity and bias the system toward specific, often maladaptive, interpretations. We use the term “stabilization” throughout this article to refer to the process by which the cognitive system selects and maintains a dominant, coherent interpretation of reality from competing possibilities. Pathological stabilization refers to a rigid or fragmented outcome of this process.

The biological embedding of this history is illustrated by epigenetic mechanisms, which can encode the effects of trauma and chronic stress via stable modifications in gene expression and can be transmitted intergenerationally (Meaney and Szyf, 2005; Yehuda and Lehrner, 2018; Yehuda et al., 2016; Franklin et al., 2010). Similarly, genetic predisposition may bias the initial setting of the precision-weighting system and its resilience to noise (Gottesman and Gould, 2003; Harrison and Weinberger, 2005). Pathological stabilization (psychosis) or hyper-stabilization (depression) may therefore arise from the interaction between trauma and a pre-existing, genetically influenced pattern of reality processing (van Os et al., 2010; Cannon, 2015). We clarify that trauma is not the sole cause of psychosis or depression, but a significant pathogenetic factor that interacts with genetic predisposition (Varese et al., 2012; Heim et al., 2008). These developmental and genetic considerations are relevant because they illustrate how early experience becomes biologically embedded and shapes the precision-weighting system, but they are not the central focus of our model.

### Research gap and objectives

Despite advances in predictive coding, dopaminergic models, and network neuroscience, a conceptual gap remains: these frameworks are rarely integrated into a single account that explains both psychosis and postpsychotic depression as related disturbances of the same underlying process. Psychosis is typically studied as a disorder of reality testing, and postpsychotic depression as a separate mood complication. The relationship between them—and the possibility that they represent opposite extremes of a single stabilization mechanism—has not been systematically developed.

This article proposes that the failure of the reality-stabilization process provides a unifying framework for understanding severe psychopathology. We specifically apply this framework to two core clinical phenomena: psychosis and post-psychotic depression. We use the term “psychosis” to refer to a syndrome characterized by a distortion of reality, which can occur in various conditions, including but not limited to schizophrenia. Schizophrenia is the prototypical example, but our model applies to psychotic processes broadly. We propose that psychosis can be conceptualized as a state of failed decoherence, where the brain cannot suppress competing perceptual and cognitive possibilities, leading to a destabilized, fragmented subjective reality. Post-psychotic depression, in contrast, may represent a compensatory hyper-stabilization, where a rigid, overly integrated—but often negatively valenced—reality is enforced to halt the chaos of psychosis, possibly mediated by altered DMN activity. We clarify that failed decoherence and hyperstabilization are not strict opposites, but two pathological extremes of the same stabilization process. Failed decoherence refers to the inability to select a dominant, coherent state, leading to fragmentation, while hyperstabilization refers to an excessively rigid fixation on a single state, leading to loss of flexibility.

Predictive coding accounts of psychosis exist (Friston, 2010; Fletcher and Frith, 2009; Corlett et al., 2011), as do phenomenological accounts of reality testing disorders (Bleuler, 1950; Andreasen, 1999). Our contribution is specific and, we believe, novel in several respects. First, we apply the quantum decoherence analogy to psychotic disintegration as a failure of cognitive selection, supported by quantum cognition research on superposition and collapse (Bruza et al., 2015; Pothos and Busemeyer, 2013; Xiong and Yu, 2025). Second, we introduce the concept of hyperstabilization as a compensatory process in post-psychotic depression. Third, we propose a unifying framework that treats psychosis and post-psychotic depression as opposite pathological extremes of the same stabilization process. Fourth, we integrate neurochemical evidence—particularly the kynurenine pathway and its modulation of glutamatergic and dopaminergic signaling—as a mechanistic substrate for stabilization failure (Tanaka et al., 2020; Tanaka and Vécsei, 2026). Fifth, we derive therapeutic implications that extend beyond dopaminergic blockade. We do not claim to replace existing models, but to connect and enrich them by placing the disruption in the act of meaning-making at the center of understanding severe mental disorders.

We will explore this hypothesis by integrating principles from predictive coding, dopaminergic modulation, network neuroscience, and the temporal dynamics of illness (e.g., Duration of Untreated Psychosis). Finally, we will derive therapeutic implications that extend beyond current dopaminergic models, aiming to promote a more functional and adaptive stabilization of reality. While we present this framework as a coherent conceptual synthesis, we emphasize that it remains hypothetical and that its main propositions require empirical testing.

### Psychosis, disorganization, and failure of stabilization

Psychosis (defined as a distortion of reality—DSM-5) can be conceptualized not as a primary presence of cognitive, positive and negative symptoms, but as a process of progressive disorganization in which the system loses its ability to reduce interference between competing possible states. We propose that disorganization represents the basic process from which both positive and negative symptoms secondarily unfold (Bleuler, 1950; Andreasen, 1999). This is a hypothesis, not an established sequence, and we present it as such.

Thus, psychosis is not only a network decoherence but a collapse of the transparency of cognition. The patient’s attention is no longer a tool they wield, but is captured by the system’s internal noise. They are forced to observe and contend with the multiple, competing realities that their brain cannot select between. The positive symptoms (hallucinations, delusions) are not just erroneous outputs; we propose that they may function as local, albeit pathological, stabilizers that the observer clings to amidst the incoherence.

The negative symptoms (avolition, alogia, blunted affect) can then be understood not merely as cognitive depletion, but as a proposed compensatory reduction in engagement. When the act of attending to reality leads only to chaos and terror, the only adaptive response may be to drastically limit the scope of attention and investment—to narrow the field of conscious engagement to a minimum, in a bid to force some stability, even if it is an impoverished one.

From the perspective of decoherence, the system fails to stabilize a dominant, coherent state after the exclusion of incompatible possibilities. Clinically, this manifests as disturbances in reality assessment, cognition, and integration of experience—not because information is missing, but because what remains after the “unloading” of interference cannot be maintained as a unified subjective reality (Uhlhaas and Mishara, 2007; Phillips and Silverstein, 2003).

The inability to stabilize the information flow leads to the depletion of cognitive and motivational resources. In the absence of a stable subjective reality, the system loses its ability to predict future states and consequences, which undermines goal-directed behavior, emotional engagement, and motivation. This process can be understood as the basis for the development of negative symptoms—which appear not as a “deficit” but as a secondary adaptation to chronic instability of experience (Strauss et al., 2014; Barch and Ceaser, 2012).

In parallel, in the presence of additional unstable and competing perceptions and interpretations, alternative, partially stabilized subjective constructions are created, which are clinically expressed as positive symptoms - hallucinations and delusions. These experiences can be seen as local attempts at stabilization in the conditions of global failure of selection and decoherence (Fletcher and Frith, 2009; Corlett et al., 2011).

We note that negative symptoms respond poorly to antipsychotic treatment compared to positive symptoms. This is consistent with our proposal that negative symptoms arise from a different mechanism—compensatory withdrawal—rather than from the same dopaminergic hyperactivity that generates positive symptoms. Antipsychotic blockade may reduce the chaos that drives positive symptoms without reversing the compensatory withdrawal that underlies negative symptoms.

At the neurophysiological level, dopaminergic dysregulation plays a key role in modulating the signal-to-noise ratio and precision distribution. A hyperdopaminergic state in the striatum leads to aberrant attribution of significance, in which incidental or irrelevant signals receive excessive weight, further destabilizing the selection of experience (Kapur, 2003; Howes and Kapur, 2009; Panov and Panova, 2024). Tanaka’s work on the kynurenine pathway provides additional mechanistic support for this framework: kynurenic acid (KYNA) modulates glutamatergic and dopaminergic signaling, and dysregulation of this pathway has been linked to both schizophrenia and psychotic symptoms (Tanaka et al., 2020; Tanaka and Vécsei, 2026). Furthermore, experimental evidence on the optimization of kynurenic acid analogs highlights how targeting specific behavioral domains through this pathway holds promising therapeutic potential for decoupling the complex symptom dimensions of schizophrenia (Martos et al., 2025). The kynurenine pathway thus represents a neurochemical substrate through which glutamatergic-dopaminergic imbalance may contribute to the destabilization of reality selection.

The therapeutic effect of D2 receptor antagonists can be understood precisely in this context: by reducing dopaminergic hyperactivity, the signal-to-noise ratio is stabilized, which alters the neurophysiological dynamics in networks involved in perception and significance. This leads to a reduction in the perceptual vitality of pathological experiences and may facilitate the restabilization of subjective reality, although often at the expense of reduced flexibility and affective intensity. We emphasize that this is an interpretation within our framework, not an established mechanism (Grace, 2016; Maia and Frank, 2017).

### Temporal progression: from cognitive impairment to deficit symptoms of a Neuroprogressive process

We propose that the clinical picture of psychosis does not appear suddenly, but develops sequentially. This progression is presented as a proposed conceptual evolution, not an established sequence. It can be rethought as an increasing depth of failure of the reality stabilization system, which follows an inversely proportional relationship: the deeper the breakdown in the basic cognitive mechanism, the more difficult the consequences may become to reverse (duration of untreated psychosis has negative consequences on prognosis) (Marshall et al., 2005; Perkins et al., 2005). We distinguish between the established association between prolonged DUP and poorer outcomes, and our interpretation that longer DUP consolidates fragmented parallel realities. Table 1 summarizes the proposed four-phase progression.

**Table 1:** Proposed four-phase progression of reality stabilization failure in neuroprogressive forms of psychosis.

Phase	Proposed mechanism	Clinical expression
Phase 1: cognitive destabilization	Unstable, labile decoherence; competing interpretations remain partially active; dopaminergic, glutamatergic, and kynurenine pathway dysregulation contribute	“Fog,” difficulty following thought or conversation, cognitive overload; forerunner symptoms
Phase 2: disorganization and negative symptoms	Failure to maintain a stable state; reduction in free energy; compensatory withdrawal to avoid exhaustion; alpha-band dysrhythmia on EEG	Disorganized thinking and behavior; avolition, blunting, apathy
Phase 3: positive symptoms as forced pseudostabilization	Over-stabilization of random internal noise; aberrant salience; hyperdopaminergic activity; coexistence with depressive and obsessive-compulsive symptoms	Hallucinations, delusions; treatment-resistant states with mixed symptom domains
Phase 4: neuroprogressive chronification and persistent deficit	Proposed structural degradation in a subgroup; provisional conceptual endpoint, not a universal trajectory	Prolonged, non-remitting cognitive deficits; resemblance to dementia process

### Phase 1: cognitive destabilization

Initially, the failure is subtle and localized in the basic cognitive stabilization tools: attention (precision) becomes unstable, working memory loses coherence, and processing speed decreases (Barch and Ceaser, 2012). We propose that this early phase corresponds to a state of unstable, labile decoherence—a condition in which the system cannot maintain a stable selection of reality, and competing interpretations remain partially active. This labile decoherence can be understood as the cognitive counterpart of the quantum decoherence analogy: just as a quantum system that has not fully decohered remains in a superposition of states, the cognitive system in this phase remains in a superposition of competing interpretations. The underlying neuropathology and neuromodulatory dysregulation—particularly involving dopaminergic and glutamatergic systems, and potentially the kynurenine pathway—may contribute to this unstable decoherence, which in turn corresponds to the initial cognitive deficit (Tanaka et al., 2020; Panov and Panova, 2024; Panov et al., 2023). At the phenomenological level, this is experienced as “fog,” difficulty following thought or conversation, and a sense of cognitive overload. These are forerunner symptoms that often precede full-blown psychosis (Fusar-Poli et al., 2013).

### Phase 2: disorganization and negative symptoms

Since the system cannot maintain a stable state, it begins to expend enormous resources in unsuccessful attempts at integration. This leads to a depletion of cognitive and motivational capacity. Clinically, this manifests as disorganized thinking and behavior (a direct result of the interference) (Uhlhaas and Mishara, 2007; Phillips and Silverstein, 2003) and negative symptoms (avolition, blunting, apathy) as a secondary adaptation (Strauss et al., 2014). We propose that this phase corresponds to a reduction in free energy by the Friston (2010) free-energy principle: the system, unable to minimize prediction error through adaptive updating, reduces its free energy by withdrawing from engagement and limiting its scope of processing. The disorganization observed clinically corresponds to dysrhythmia in the alpha frequency band on EEG, which has been associated with treatment-resistant schizophrenia, disorganized symptoms, and dissociation (Panov et al., 2026). This alpha dysrhythmia may reflect the underlying failure of neural coherence that accompanies the cognitive fragmentation. We propose that these symptoms represent an energy conservation strategy - the system reduces its attempts at stabilization to avoid total exhaustion.

### Phase 3: positive symptoms as forced pseudostabilization

In its desperation, the system begins to “catch” and overly stabilize random bits of internal noise or misinterpretations. These are hallucinations and delusions—we propose that they may represent local, albeit pathological, stabilizers in a sea of disorganization (Fletcher and Frith, 2009; Corlett et al., 2011). To the observer (the patient) they are terrifying, but to the system itself they may provide some structure, albeit a pathological one. From a neurophysiological perspective, this state is facilitated by hyperdopaminergic activity, which gives aberrant significance to random signals (Kapur, 2003; Howes and Kapur, 2009). We further propose that disorganized symptoms are closely linked to positive symptoms and to the development of depressive and obsessive-compulsive symptoms, as observed in treatment-resistant psychotic states. In such states, positive, negative, depressive, and obsessive-compulsive symptoms often coexist, suggesting a shared underlying failure of stabilization (Panov, 2022; Panov et al., 2024). We propose that positive symptoms may often coincide with or follow negative symptoms because they are the product of the same underlying instability.

### Phase 4: neuroprogressive chronification and persistent deficit

If the failure to stabilize is prolonged and profound the system may undergo structural degradation. We propose that the persistent inability to form coherent mental representations may lead to a reduction in integrative capacity. The neural networks responsible for this function may disintegrate through inactivity or chaotic activity. Clinically, this manifests itself in prolonged, non-remitting cognitive deficits that resemble a dementia process (e.g., Kraepelin’s “dementia praecox”) (Bleuler, 1950; Panov et al., 2023). We note that Bleuler himself moved away from the dementia praecox concept, and that current evidence suggests that most cognitive impairment in schizophrenia is present at or before the first episode, with no clear further decline afterward (Bora and Murray, 2014; Heilbronner et al., 2016). We further note that the clinical course of schizophrenia is heterogeneous. Meta-analytic evidence indicates that cognitive deficits remain largely stable after the first episode in most patients, with no clear evidence of continued neurodegenerative decline (Demirlek et al., 2026; Ghanem et al., 2025). However, a subgroup of patients with severe, pervasive cognitive impairment may show a more pronounced trajectory (Catalan et al., 2024). We therefore present this phase as a provisional conceptual endpoint that may apply to a specific subgroup, rather than as a universal progression.

### Postpsychotic depression and hyperstabilization

Postpsychotic depression can be viewed as a phenomenon of hyperstabilization following a psychotic episode. On the one hand, it creates a coherent and relatively integrated subjective reality that reduces the chaos of parallel and fragmented perceptions characteristic of psychosis. We propose that depression can be interpreted as a “sought” effect: by stabilizing the system, it provides a basis for adaptation to an integrated social reality, albeit often with limited adaptive potential for the individual.

This process has been hypothesized to involve hyperactivity of the default mode network (DMN), which supports autobiographical and self-referential processes, allowing the integration of fragmented experiences that occur during psychosis (Sheline et al., 2009; Hamilton et al., 2011; Greicius et al., 2007; Kaiser et al., 2015). However, direct evidence for DMN hyperactivity in postpsychotic depression is limited and mixed. In major depression, DMN hyperconnectivity is a relatively consistent finding (Greicius et al., 2007; Kaiser et al., 2015), but in remitted psychotic depression, Neufeld et al. (2018) found decreased between-network connectivity between the DMN and salience/insular regions. This suggests that DMN-related abnormalities in psychotic depression may not simply reflect hyperactivity, and that further research is needed to clarify the direction of DMN changes in this population. We therefore present the DMN hyperactivity hypothesis as tentative and in need of direct empirical testing in postpsychotic populations.

### Relationship with the negative symptoms of psychosis

The clinical picture of postpsychotic depression—characterized by loss of energy (anergia), inability to experience pleasure (anhedonia), emotional blunting, and reduced expression—shows a striking resemblance to the negative symptoms of psychosis itself. We propose that this resemblance reflects a shared origin and functional purpose: hyperstabilization of the system at the expense of its plasticity and affective range. However, clinical similarity does not by itself establish an identical functional origin. The shared mechanism remains a hypothesis. The key difference lies in the temporal dynamics and the triggering context. In the negative symptoms within active psychosis, hyperstabilization (the apathetic character) appears as an early, often protective reaction against the escalating process of disorganization. It represents an energy conservation strategy and a forced reduction of cognitive volume to prevent the complete collapse of reality. In postpsychotic depression, hyperstabilization follows the psychotic episode. It is a reaction to the experienced chaos and aims to prevent relapse by imposing an extremely rigid, pessimistically colored, yet consistent model of reality. Therefore, the two processes share an identical clinical manifestation, reflecting a fundamental reduction in exploratory activity and emotional engagement with the world—the price paid for restoring order (Conley et al., 2017; Galderisi et al., 2018).

On the other hand, the development of depressive symptoms is often closely linked to a previous state of anxiety, which can be conceptualized in the framework of predictive coding. Anxiety leads to generalized negative expectations about the future, with overprocessing and exaggerated significance of potential threats leading to the perception of catastrophic consequences as probable and realistically possible. This creates a deep sense of inevitability and catastrophic loss, leading to depressive withdrawal and clinical expression of postpsychotic depression (Barrett and Simmons, 2015; Paulus and Stein, 2006). We present this as a proposed causal pathway, not an established sequence.

Thus, postpsychotic depression can be understood as a two-component process: on the one hand, a stabilizing hyperstabilization of network “chaos”; on the other hand, a negative expectation generated by previous anxiety and predictive coding, leading to passivity, alienation, and emotional inhibition.

### Relationship with DUP and temporal dynamics

The duration of untreated psychosis (DUP) is of key importance for understanding the development of psychotic and postpsychotic processes. A longer period of decoherence allows the accumulation and consolidation of fragmented parallel realities and progressively makes it more difficult to stabilize subjective reality. This affects cognitive, emotional, and motivational integration, increasing the risk of chronic disorganization and enhancing the development of secondary negative, positive and depressive symptoms (Marshall et al., 2005; Perkins et al., 2005). We distinguish the established association between prolonged DUP and poorer outcomes from our interpretation that longer DUP consolidates fragmented parallel realities.

From a clinical perspective, DUP is an indicator of the temporal dynamics of psychosis, which largely determines the consolidation of the “stability” of chaotic and fragmented parallel perceptions and further complicates the interference toward an integrated network configuration. Early intervention, through medication and psychosocial interventions, allows for a faster restoration of the coherence of the system and minimizes the negative consequences for the individual’s adaptation to the social environment. In this context, the therapeutic approach should take into account not only the momentary stabilization through dopaminergic antagonists, but also the temporal dynamics of the processes of integration and decoherence. Concretely, this means that interventions should be timed to the phase of illness: early intervention aims to prevent consolidation of fragmented realities; later intervention focuses on restoring coherence and preventing relapse.

## Therapeutic implications beyond dopamine

### Psychosocial and cognitive-behavioral interventions

If psychosis stems from a failure to integrate experience into a stable reality, then therapies facilitating this integration are paramount.

Cognitive Behavioral Therapy for psychosis (CBTp) helps patients relatively-test and re-evaluate aberrant beliefs (delusions) and perceptions (hallucinations), effectively recalibrating the precision-weighting of internal models against sensory evidence (Hofmann et al., 2012; Turner et al., 2020). Furthermore, metacognitive training directly targets the cognitive biases (e.g., jumping to conclusions) that maintain fragmented realities, fostering greater cognitive flexibility and doubt in overly rigid interpretations (Moritz et al., 2014). On a narrative level, trauma-focused therapies address the fragmented and adverse prior experiences that corrupt the “possibility space” from which reality is selected, allowing for the integration of traumatic memories into a coherent autobiographical narrative, which may reduce their intrusive and destabilizing power (van den Berg et al., 2016).

### Targeting network coherence and neuroplasticity

Therapeutic strategies can aim to directly influence the neural substrates of reality stabilization. Cognitive remediation therapy (CRT) is designed to improve core cognitive functions—such as working memory, attention, and cognitive control—that are the very tools of stabilization. By strengthening these capacities, CRT may enhance the brain’s ability to select and maintain coherent cognitive states (Wykes et al., 2011). Emerging neuromodulation techniques like transcranial direct current stimulation (tDCS) are being investigated for their potential to modulate cortical excitability and network dynamics, potentially favoring a rebalancing of the dysfunctional DMN-SN interplay implicated in both psychosis and depression (Brunoni et al., 2014; Mondino et al., 2018). We note that these links are interpretive and that direct evidence for restoration of DMN-SN balance through these interventions is limited.

### Epigenetic and lifestyle considerations

The epigenetic dimension of our framework suggests that interventions promoting a salutogenic environment can support the biological basis for stabilization. Reducing chronic stress through mindfulness-based interventions and improved social support may help normalize dysregulated stress-response systems (e.g., HPA axis) whose alterations are epigenetically embedded and contribute to cognitive fragmentation (McEwen and Gianaros, 2010; Hölzel et al., 2011). Furthermore, physical exercise and environmental enrichment are potent stimulators of neuroplasticity and neurogenesis, particularly in the hippocampus—a key region for memory integration and contextual processing—and may support the brain’s intrinsic capacity for adaptive reorganization (Firth et al., 2017; Nithianantharajah and Hannan, 2006).

In general we can say that moving “beyond dopamine” means adopting a multi-level rehabilitation model of the reality-stabilization apparatus. This model synergistically combines pharmacological agents to establish a basic signal-to-noise ratio, with psychosocial and cognitive tools to repair the selection algorithms, and lifestyle interventions to foster a neurobiological milieu conducive to integration and adaptive plasticity.

The dynamics in the development of the psychotic process also provide grounds for a temporal therapeutic approach, which we present as a provisional framework, not a prescriptive sequence. In the early phase (cognitive), the goal is rapid stabilization through medication combined with strengthening of cognitive tools through cognitive rehabilitation, in order to stop the progression at the root. In the acute phase (positive/negative), the goal is to reduce parasitic stabilizations through antipsychotics and to maintain motivation through behavioral activation. In the chronic phase (neuroprogressive), the goal shifts to compensatory strategies and a supportive environment. Concretely, this may include structured daily routines, social skills training, supported employment, family psychoeducation, and cognitive aids, rather than assuming that the underlying integrative capacity can be restored.

We also acknowledge that many patients do not respond well to these treatments, or to antipsychotics. Within our framework, this may reflect the depth of the stabilization failure, the rigidity of the hyperstabilized state, or the presence of additional factors (e.g., trauma, social adversity) that are not addressed by the interventions described. This remains an open question for future research.

### Limitations

The framework proposed here remains hypothetical, and several limitations should be acknowledged. First, there is no direct empirical evidence for decoherence-like processes in the brain; the quantum analogy is structural, not literal. Second, the DMN literature in depression is mixed, with some studies reporting reduced connectivity and others increased connectivity, and direct evidence in postpsychotic populations is limited. Third, the relationship between negative symptoms and postpsychotic depression remains unclear; clinical similarity does not by itself establish an identical functional origin. Fourth, the four-phase progression is a proposed conceptual model, not an established trajectory, and should allow for variability in illness trajectories. Fifth, the therapeutic implications described here are interpretive and require empirical validation. Future work should focus on empirical testing of stabilization and hyperstabilization mechanisms, longitudinal assessment across illness stages, and their possible therapeutic relevance.

## Conclusion

We have presented a unifying framework that views psychosis and postpsychotic depression as opposite extremes of the same fundamental process: the stabilization of subjective reality. By analogy with quantum decoherence and predictive coding mechanisms, we propose that psychosis represents a failure of cognitive selection and integration, leading to disorganization and “parasitic” stabilizations (positive symptoms). In contrast, postpsychotic depression is a compensatory hyperstabilization—a rigid contraction of reality around a single, often negative, picture, possibly mediated by altered DMN activity, although direct evidence in postpsychotic populations remains limited and mixed.

We propose that this perspective may help explain the natural history of the disease, including the temporal progression from cognitive deficits to disorganization and finally to positive symptoms and deficit symptoms, as a consequence of the increasing depth of failure of the stabilizing apparatus. The therapeutic implications are potentially important: the goal shifts from suppressing isolated symptoms to restoring adaptive capacity for stabilization. This requires an integrated approach, combining pharmacological modulation of precision (dopamine) with cognitive rehabilitation of the tools of attention and integration, as well as therapeutic work on traumatic experiences that distort the very “field of possibilities.”

As such, the reality stabilization framework does not replace, but rather connects and enriches existing biological and psychological models. It offers a framework that may complement existing models by placing the disruption in the very act of meaning-making at the center of understanding severe mental disorders, opening avenues for more holistic and etiologically informed forms of intervention.

This article proposes that psychosis and postpsychotic depression can be understood as opposite pathological extremes of a single process: the stabilization of subjective reality. Psychosis is framed as a failure of cognitive selection and integration—a failure of decoherence—leading to fragmentation and parasitic stabilizations. Postpsychotic depression is framed as a compensatory hyperstabilization. The framework remains hypothetical, and its main propositions require empirical testing. Key uncertainties include the lack of direct evidence for decoherence-like processes, the mixed findings on DMN connectivity, the unclear relationship between negative symptoms and postpsychotic depression, and the provisional nature of the four-phase progression.

## Data Availability

The original contributions presented in the study are included in the article/supplementary material, further inquiries can be directed to the corresponding author/s.
